# Effects of Adaptations in an Interdisciplinary Follow-Up Clinic for People with Spinal Cord Injury in the Chronic Phase: A Prospective Cohort Study

**DOI:** 10.3390/jcm12247572

**Published:** 2023-12-08

**Authors:** Julia Tijsse Klasen, Tijn van Diemen, Nelleke G. Langerak, Ilse J. W. van Nes

**Affiliations:** 1 Department of Rehabilitation Medicine, Radboud University Medical Center, 6525 GA Nijmegen, The Netherlands; julia.tijsseklasen@ru.nl (J.T.K.); i.vannes@maartenskliniek.nl (I.J.W.v.N.); 2 Department of Spinal Cord Injury Rehabilitation, Sint Maartenskliniek, 6500 GM Nijmegen, The Netherlands; 3 Department of Research, Sint Maartenskliniek, 6500 GM Nijmegen, The Netherlands; n.langerak@maartenskliniek.nl

**Keywords:** spinal cord injuries, follow-up care, ambulatory care, prevention, interdisciplinary, secondary health conditions

## Abstract

People with spinal cord injury (SCI) often experience secondary health conditions (SHCs), which are addressed during interdisciplinary follow-up clinics. We adapted the design of our clinic, by introducing a questionnaire concerning functioning and SHCs, additional measurements of blood pressure and saturation, and participants were seen by either a specialized nurse or rehabilitation physician. In this study, we investigated the effects of these adaptations and the experienced satisfaction of the participants. The results showed an increased number of recommendations in the adapted design, compared to the initial design. Further, the nature of the recommendations shifted from somatic issues to recommendations regarding psychosocial functioning and regarding (the use of) devices. The added measurements revealed an average high systolic blood pressure, which led to more referrals to the general practitioner. The clinical weight and pulmonary functions stayed stable over time. The current adaptations in design expanded and optimized the number and nature of recommendations regarding SHCs to participants. The questionnaire helps the participant to prepare for the clinic and the professionals to tailor their recommendations, resulting in highly satisfied participants.

## 1. Introduction

A spinal cord injury (SCI) is damage to the bundle of nerves and nerve fibers that sends and receives signals from the brain. The spinal cord extends from the lower part of the brain down through the lower back [1]. This damage can cause temporary or permanent changes in feeling, movement, strength and body functions below the site of the injury [2]. Along with such an injury, people with SCI could also have secondary health conditions (SHCs) such as bladder and bowel problems, respiratory issues and skin issues [3].

These SHCs occur in both the physical and mental domains and might cause an additional burden and restrictions in participation [4,5,6]. In a large study examining the occurrence of SHCs, an average of eight different SHCs were found, for each person with an SCI [5]. Both physical and mental SHCs can influence quality of life and participation and lead to increased medical consumption with higher costs [3,7,8,9,10]. In an effort to reduce medical costs and because of the scarcity of health professionals, the Dutch government encourages various vulnerable groups to rely on formal and informal caregivers in order to enable them to live longer independently [11].

For primary-care professionals, providing care for people with an SCI can be challenging, especially when there is a lack of sufficient knowledge about this relatively rare disorder, which has an annual incidence of 40–80 cases per million [12]. Physicians in the rehabilitation centers specialized in SCI possess expertise, but they are no longer primarily responsible for medical care in the home situation [13].

In the Netherland s, there is a guideline in accordance with the Dutch and Flemish Spinal Cord Association [14], which emphasizes the need for interdisciplinary (bi-)annual follow-up care for people with SCI, focused on the prevention, monitoring and treatment of SHCs and to support the informal and primary caregivers.

In 2005, an interdisciplinary follow-up clinic for people with SCI was established at the Sint Maartenskliniek, the Netherlands. The main goal of this interdisciplinary follow-up clinic was to promote healthy aging of people with SCI. This goal is met by giving recommendations to the participants and the primary healthcare providers on how to prevent new SHCs or to control existing SHCs. This follow-up clinic is organized in a carrousel model, in which a participant visits five different disciplines within one appointment at the rehabilitation center. The therapeutic content of this follow-up clinic was investigated in an explorative retrospective study based on data between January 2012 and October 2020. That study showed that an interdisciplinary follow-up clinic can result in a wide and extensive range of recommendations for participants of these clinics [15]. Further, that same study recommended screening for SHCs prior to the interdisciplinary follow-up clinics, to be sure to cover all possible SHCs and tailoring the recommendations to improve healthcare [15].

In October 2020, based on these recommendations [15], adaptations were made in the design of the interdisciplinary follow-up clinic at Sint Maartenskliniek. To help participants of the interdisciplinary follow-up clinic think about all the possible changes and health problems and to prepare the team, a questionnaire was sent to the participants prior to their appointment, concerning the physical, mental and social wellbeing, as well as questions about practical aspects (e.g., use and state of assistive devices). The second adaptation to the interdisciplinary follow-up clinic was to add the measurement of blood pressure, oxygen saturation and heart rate during the visit. Further, the visit to the physician and the specialized nurse were combined and carried out by only one of the two disciplines. This was more efficient, since the participants experienced substantial overlap between the two professions in the initial follow-up clinic. Lastly, the satisfaction of the participants with the setup of the clinic was investigated. With these adaptations in the protocol, the professionals of the team gained more information about the participants, which was thought to result in better and more personalized recommendations after the follow-up visit.

The aim of this study was to investigate the impact of the changes made to the interdisciplinary follow-up clinic by (1) determining whether there was a change in the number and nature of recommendations given to participants with SCI between their visit to the initial interdisciplinary follow-up clinic and their more recent visit with the adapted design; (2) investigating the value of the physical outcomes; (3) investigating the value of the newly introduced questionnaire prior to the clinic; and (4) investigating the satisfaction of the participants with the adapted design and the given recommendations.

We hypothesized that the adapted follow-up care design, with more collected information, will contribute to more personalized recommendations leading to prevention of SHCs in the future and participants being more satisfied with their follow-up care.

## 2. Materials and Methods

Design and setting: This is a prospective cohort follow-up study of participants attending an interdisciplinary follow-up clinic for people with SCI conducted at the Sint Maartenskliniek in Nijmegen, one of the eight specialized SCI rehabilitation centers in the Netherlands. Participants were actively identified from electronic patient files.

Participants: People were eligible for this study if they were 18 years or older, had a diagnosis of SCI and visited one of the interdisciplinary follow-up clinics between October 2020 and October 2022. In general, people with SCI were considered for participation in the interdisciplinary follow-up clinic when they had a motor complete SCI, were wheelchair users, or had other complex physical conditions which might benefit from an interdisciplinary approach [15].

Procedure: After being invited to the interdisciplinary follow-up clinic, an electronic questionnaire was sent to the participant three weeks before the actual visit. If there was no e-mail address available or the participant preferred a paper version, this was sent by mail. Participants were asked to complete and return the questionnaire via mail or send the paper version back, so that the information could be up-loaded to the electronic patient file. All members of the interdisciplinary follow-up clinic had access to the completed questionnaire in order to prepare themselves for the clinic. On the interdisciplinary follow-up clinic day, five participants came to the Sint Maartenskliniek, where they started at the same time and visited five stations consisting of a rehabilitation physician or a specialized nurse, a physiotherapist, an occupational therapist, a psychologist or social worker and a nurse in a random order. At the end of the interdisciplinary follow-up clinic, the participants were asked for permission to use their anonymized data for research purposes. Further, they were asked to rate their satisfaction regarding the adapted interdisciplinary follow-up clinic. After the interdisciplinary follow-up clinic, the team members had a debriefing where all the recommendations to the participants were gathered and classified by one of the team members to one of the categories as determined and described in a previous retrospective study [15]. These recommendations were communicated to the participants by phone; their general practitioner and other involved healthcare professionals received a letter, including all recommendations and findings.

In line with the previous study [15], the nature of the recommendations was divided into four domains: preventative to be applied at home, treatment at the SCI department of Sint Maartenskliniek (internal treatment), treatment in a hospital or by a primary healthcare professional (external treatment), or (medication) prescription. In each of these four domains, the recommendations were divided into thirteen categories including pain, spasm, skin problems, bowel control problems, bladder control problems, lung problems, all other intercurrent physical/medical problems, splints, devices, social problems, psychological problems, seating advice and functioning.

The data were collected using Castor EDC (version 2022.1.0.3) (EU HQ, Amsterdam, The Netherlands).

Outcome measures: Data were based on demographic and SCI characteristics, a questionnaire completed by the participants prior to the interdisciplinary follow-up clinic visit, physical assessments during the clinic, a list of recommendations from the clinicians, and a questionnaire about participants’ satisfaction with the set-up of the adapted interdisciplinary follow-up clinic.

Data collected from the participants’ medical records included demographic characteristics, time since injury, level of SCI, completeness of SCI and cause of SCI.

The questionnaire was a combination of different outcome measures. This included some validated outcome measures, the Spinal Cord Injury Secondary Conditions Scale (SCI-SCS), International Spinal Cord Injury Quality of Life basic dataset (QoL-BDS), the Patient Health Questionnaire-2 (PHQ-2) and the Generalized Anxiety Disorder-2 (GAD-2). The selection of the validated measures was in close collaboration with all Dutch specialized SCI rehabilitation centers. While this took place during the inclusion period, some measures were added during the process of data collection. Further, the questionnaire consisted of some self-developed structured questions to gather information about current functioning.

The SCI-SCS [16] consists of 16 items, each addressing one health condition. Participants rate on a 4-point scale how much each health problem affected their activities and independence in the last three months, ranging from 0, not a problem, to 3, significant or chronic problem, resulting in a score between 0 and 48 [16]. Two extra questions were added to the SCI-SCS regarding sleeping problems [17] and weight problems. The response categories were the same.

The International Spinal Cord Injury Quality of Life basic dataset (QoL-BDS) consists of four questions regarding satisfaction with quality of life as a whole, with physical health, psychological health and with social life. Each question is rated on a 0–10 numeric rating scale. The total score is the average of the four items resulting in a range of 0–10 [18].

The PHQ-2 is the two item version of the original PHQ-9 [19]. The first two questions of the original scale were used to screen for basic symptoms of a depressive disorder according to the Diagnostic and Statistical Manual of Mental Disorders, 4th Edition [20]. The two questions are scored on a scale ranging from 0, not at all, to 3, nearly every day, resulting in a range of 0–6 [21].

The GAD-2 is the short screening version of the GAD-7 [22]. The first two questions of the original scale are used to screen for the basic symptoms of generalized anxiety and are scored on a scale ranging from 0, not at all, to 3, nearly every day, resulting in a combined total range of 0–6 [23].

The self-developed structured questionnaire started with seven questions focusing on information about the current functioning and the use of assistive devices. This was followed by eight questions about changes in physical functioning in daily life. An example of a question is the following: Since your last visit to the rehabilitation clinic, has there been any change in your walking, standing, or transfers? A total of eleven questions investigated changes in participation and social activities. An example of a question is the following: Since your last visit to the rehabilitation center, are there any problems with respect to leisure time or hobbies?

The physical assessment conducted by the nurse included clinical weight, blood pressure, oxygen saturation, heart rate and other physical signs, e.g., prevalence of pressure injuries and wounds. Assessments conducted by the physiotherapist included pulmonary function (forced vital capacity (FVC), peak expiratory flow (PEF), forced expiratory volume in 1 s (FEV_1_)).

After the follow-up clinic visit, participants were asked to complete a questionnaire about their satisfaction with the set-up of the adapted interdisciplinary follow-up clinic. They were asked to what extent their expectations were met and whether their questions were answered. Answers were scored on a numeric rating scale, ranging from 0, very unsatisfied/not at all, to 10, very satisfied/completely.

Statistical analyses: Descriptive statistics were performed for all collected variables. Continuous data were presented as mean values with standard deviations and ranges (minimum–maximum). Categorical data were presented using counts and percentages.

When applicable, the gathered data included participants’ last visit to the initial design (recommendations and some physical measurements), which were compared to the data of their first visit with the adapted design. The change in the number of recommendations given to the participants per domain and per category were analyzed using paired sample *t* tests. Due to repetitive measurements, a level of significance of *p* < 0.001 was used.

For calculating (sub)scale scores of the validated measures, missing items were replaced with the mean score of that scale if the total extent of missing items was less than 20%, otherwise the (sub)scale was considered missing [24].

To determine the relationships between the total amount of problems identified by the participants (based on the validated outcome measures of the questionnaire completed prior to the follow-up clinic visit) and the total number of recommendations provided by the professionals, Pearson product-moment correlation coefficients were calculated. *p*-values less than 0.05 were considered statistically significant. Correlations up to 0.3 were considered as weak, between 0.3 and 0.5 as moderate and above 0.5 as strong [25].

All analyses were conducted using SPSS for Windows (version 27) (IBM Corp, Armonk, NY, USA).

## 3. Results

### 3.1. Background

A total of 204 participants visited one of the 52 adapted interdisciplinary follow-up clinics between October 2020 and October 2022. Nine participants did not give consent and were excluded from the study.

Of the 195 participants, 56 did not visit the initial interdisciplinary follow-up clinic [15], and therefore, they were excluded from the analysis regarding the comparison between the two designs. The characteristics of the 195 and 139 participants are shown in Table 1.

### 3.2. Recommendations

**Figure 1:**
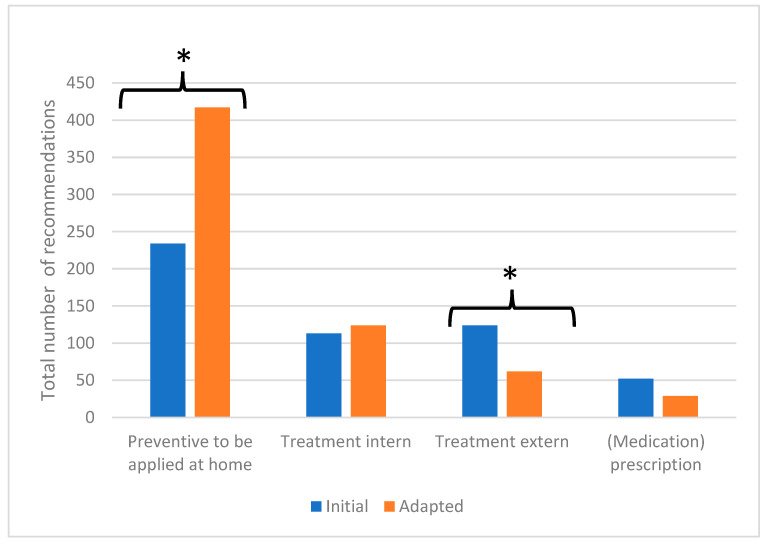
Total number of recommendations in four domains given in both designs of the follow-up clinic. (N = 139, * *p* < 0.001 according to a paired samples *t*-test).

For the 139 participants that visited both designs, a total of 523 recommendations were given in the initial design and 628 recommendations in the adapted design. This resulted in a significant increase in the average number of recommendations from 3.7 to 4.6 per participant. Figure 1 shows the total number of recommendations given to the 139 participants at both follow-up clinics in each of the four domains. Most recommendations were preventive in nature and could be applied at home, with 45% (*n* = 234) of the total number of recommendations in the initial design increasing to 66% (*n* = 417) in the adapted design (*p* < 0.001). A decrease in the number of recommendations was made regarding external treatment, which went from 24% (*n* = 124) in the initial design to 10% (*n* = 62) in the adapted design.

Table 2 shows the distribution of the recommendations per domain over the different categories for the initial and the adapted interdisciplinary follow-up clinic.

Most of the recommendations in the initial design (*n* = 523) were related to medical intercurrent (27%), while in the adapted design (*n* = 628), most recommendations were related to devices (19%), closely followed by functioning (18%). An increase in the number of recommendations was observed regarding social and psychological problems, preventive at home, which went from 2% and 2.5% in the initial design to 6% and 5% in the adapted design (*p* < 0.001), respectively.

Over the four domains there was an increase in the number of recommendations regarding lung problems, social problems, devices and seating advice, while recommendations about medical intercurrent and bladder control problems decreased.

### 3.3. Physical Assessments

Outcomes of the physical assessments of the 139 participants who visited both designs of the follow-up clinic are shown in Table 3. Not every physical outcome was measured consistently during the follow-up clinic, which resulted in some missing data.

The total number and percentage of pressure injuries or wounds present remained consistent as well as the measurements of pulmonary functions, even though these functions were measured in fewer participants, 102 in the initial design and 86 in the adapted design. Due to COVID-19 regulations applied during part of the measurement period, only participants who were expected to be at high risk for pulmonary deterioration were measured.

The added cardiopulmonary measurements In the adapted design showed, on average, an oxygen saturation within the normal range (95–100%).

Concerning the systolic blood pressure, 55 participants who had taken part in both designs had a systolic pressure of ≥140 mm Hg, of which, 28 had a systolic pressure of ≥160 mm Hg. Of these 28 participants, 20 had a complete SCI (AIS A) and 11 had a level of SCI of Th6 or above. The average age and weight among these 28 participants were, respectively, 65 years (SD = 11.9) and 82.1 kg (SD = 15.9). The highest systolic pressure measured by a participant was 220 mm Hg.

The average heart rate of 74 BPM (SD = 14.9) was within the normal range (60–100 BPM).

### 3.4. Questionnaire

Table 4 shows the scores of the validated outcome measures completed prior to the interdisciplinary follow-up clinic.

**Figure 2:**
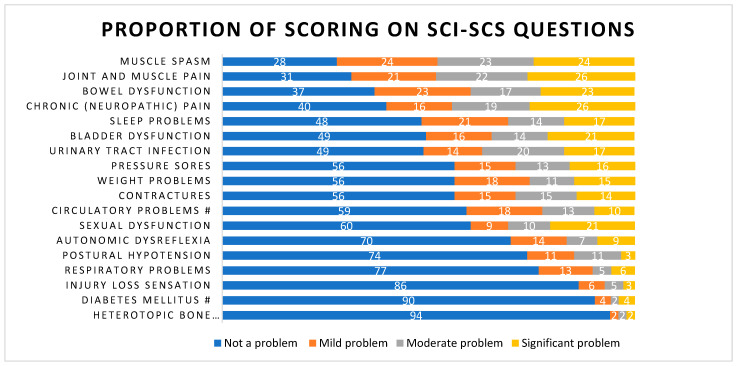
Proportions (%) of scorings on the separate questions of the Spinal Cord Injury Secondary Condition Scale. N = 176. Abbreviations: SCI-SCS, Spinal cord injury secondary condition scale. # N = 103 because the questions were added later.

The score distribution on the separate questions of the SCI-SCS, including the two added questions regarding sleep problems and weight problems, is shown in Figure 2. On average, every participant indicated at least mild problems with six different SHCs on the original SCI-SCS and seven on all 18 questions used in this study. The SHCs reported by more than half of the participants as at least a mild problem were muscle spasms, joint and muscle pain, bowel dysfunction, chronic neuropathic pain, sleep problems, bladder dysfunction and urinary tract infections.

The mean scores on the four Qol-BDS questions range between 6.4 (for physical health) and 7.1 (for psychological and social life).

With regard to PHQ-2 and GAD-2, a total of 30% and 29% participants scored two or higher, respectively, indicative of problems with depressive mood or anxiety [26,27].

A moderate association was found between the total number of recommendations and the SCI-SCS, and weak but significant associations were found with quality of life associated with physical health and the anxiety score of the GAD-2.

In the questionnaire, 87 of 180 participants indicated that they were receiving therapy in their home environment. Of those, eighty-five visited a physiotherapist, seven an occupational therapist, four a psychologist, two a social worker and two another healthcare professional. Of the eight questions regarding changes in physical functioning since their last visit, the participants on average answered positively on 1.4 (SD = 1.6, range 0–6) questions. On average, the participants positively answered 3.9 (SD = 2.2, range 0–11) out of eleven questions regarding changes in their participation and social activities. Note that part of the study period was during substantial social restrictions due to the COVID 19 pandemic.

### 3.5. Satisfaction with Adapted Design

A total of 191 participants completed the three questions after the interdisciplinary follow-up clinics. They indicated their satisfaction with the set-up of the adapted clinic as 8.8/10 on average. In addition, when asked to what extent their expectations were met and their questions were answered, they scored 8.9/10 and 9.0/10, respectively.

## 4. Discussion

The objective of this study was to investigate the value of adaptations to the design of an interdisciplinary follow-up clinic for people with SCI, by studying the change in the number and nature of recommendations, the value of physical measurements, the value of a questionnaire completed prior to the clinic and the participants’ satisfaction with the adapted follow-up clinic.

Based on 139 participants who visited both designs of the interdisciplinary follow-up clinics, there was a significant increase in the average number of recommendations per participant. The increase in recommendations is related to more recommendations regarding devices and functioning, especially those that are preventive in nature. This increase could indicate an effect of the addition of the questionnaire, because this questionnaire draws attention to problems with facilities and devices, as well as psychosocial issues. In these three categories, there is an increase in the number of recommendations given to the participants. In general, when visiting health care providers, people tend to focus on somatic and physical discomfort, rather than mental or social issues [28]. Nevertheless, problems regarding social relationships and financial strain could contribute to the mental health burden of people with SCI [29]. Given these results, one could argue that the questionnaire ensures that problems regarding devices and psychosocial aspects become more insightful and thus easier to discuss. Because of the wide range of topics in the questionnaire, the participant might be primed to ask certain questions more easily during their visit to the interdisciplinary follow-up clinic, however, this might also result in decreased attention to issues of other categories.

The shift in nature of the recommendations may be due to the change in the clinic design, where the participant is now seeing either a rehabilitation physician or a specialized nurse but not both, resulting in less time to discuss all physical aspects related to the SCI. Constant attention for all physical and mental SHCs is essential for comprehensive follow-up care for all people with SCI. The decrease in the number of recommendations regarding bladder control needs further consideration and development of interdisciplinary follow-up clinics.

The decrease in the number of recommendations regarding intercurrent medical issues could be due to the changed method of registering the recommendations of the follow-up clinic. Although the same domains and categories were used in both studies, in the adapted design, the recommendations were classified by one of the team members during the debriefing, while in the initial design, the researcher had to subdivide the recommendations retrospectively from the letters to the general practitioner. This new method of registering may provide clearer categorization for recommendations as opposed to being classified as intercurrent medical issues.

People with SCI, particularly those with a high level of SCI, tend to have lower blood pressure in general and are more likely to have episodes of orthostatic hypotension [30]. However, the participants in this study had an, on average, high, mainly systolic blood pressure. The high systolic blood pressure might be caused by the so-called “white-coat-effect”. As a British study mentions, this effect should not be overlooked, and with certain systolic pressures, it might be necessary to carry out an ambulatory, repetitive measurement, in order to gain a more realistic view of one’s blood pressure [31]. Other factors can contribute as well, such as stress or the discomfort of getting to the follow-up clinic, especially for participants with higher spinal cord lesions. Also, the natural changes in blood pressure that can occur during the day, which more often occurs in people with SCI, could be an explanation for these findings [32]. The last contributing factor could be the fact that screening for high blood pressure does not take place adequately in people with SCI in general. When a physician is lacking sufficient knowledge about SCI, this might result in the delayed diagnosis and treatment of secondary health conditions, the so called doctor’s delay. The findings in this study show that a one-time measurement during a participants’ visit does not properly represent these possible patterns of blood pressure. All these contributing factors argue in favor of performing ambulatory/repeated blood pressure measurements, in order to distinguish between a one-time high systolic blood pressure or a structural problem. For this reason, more participants were referred to their general practitioner for regular check-ups.

Due to coronavirus, the measurement of the pulmonary function was restricted during part of the inclusion period. During these restrictions, only participants who were at risk for deterioration of their pulmonary function were measured. Despite these restrictions, there was no significant difference in the outcomes of the pulmonary function of the participants in the adapted design, consistent with previous findings from the retrospective study. This is consistent with retrospective findings of the initial design of the interdisciplinary follow-up clinic [15]. Notwithstanding the fact that pulmonary function remained stable over time, it is important for the wellbeing of the participants and to prevent deterioration to consistently monitor and conduct these measurements in order to give adequate recommendations with regard to their pulmonary function.

The total number of recommendations given to the participants shows a moderate positive correlation with the total score on the SCI-SCS. The more impact participants experience from different SHCs, the more recommendations they received during the interdisciplinary follow-up clinic. For the other validated scales, there was only a weak correlation; although for the QoL-BDS question regarding their physical health and the anxiety scale, the correlations were significant. The lack of a strong association between these scales and the total number of recommendations does not diminish their value. They might help provide the team members with a more comprehensive understanding of the participant’s situation, which could further assist in tailoring the recommendations.

On the extended SCI-SCS (18 items), the participants indicated that they have problems with an average of seven different SHCs at the time of the interdisciplinary follow-up clinic. This is comparable with a large cross-sectional study in the Netherlands regarding SHCs, although the instruments used differ [5]. The average amount of SHCs on the original SCI-SCS (16 items) is the same as found in the validation and in another study with the SCI-SCS [16,33]. The distribution of the scoring on the different SCI-SCS questions is somewhat different between the different studies [8,16,33,34]. The differences found between the studies might be due to differences in the inclusion criteria, the time since injury and age of the participants investigated. The additional questions about sleep and weight problems appear valuable as almost half or more than half of the participants indicate weight problems or sleeping problems, respectively.

The scores on the QoL-BDS are very similar to the scores in an international clinometric study of the QoL-BDS [18]. Part of the inclusion period of the current study was during substantial social restrictions due to the COVID 19 pandemic, many participants stated that their score on the QoL-BDS social life was lower at that time than normal. Nevertheless, the average score on the QoL-BDS social life is comparable data from an international study gathered before the pandemic.

On the total PHQ-2 in this sample, 70% of the participants scored 0 or 1 indicating that there likely was no depressive mood problem [26]. This 70% which are not likely to have mood problems is still 10% less than a non-depressed community based sample [21].

The participants’ satisfaction scored very high on average, which shows that participants considered this follow-up clinic of added value. These findings are consistent with previous research [35]. Participants saw value in the follow-up clinic and as a way to answer their questions regarding their SCI and to prevent or follow up on SHCs. The time invested in completing the questionnaire most participants was not a big investment, as supported by the very high ratings concerning satisfaction with the set-up of the interdisciplinary follow-up clinic.

The willingness to participate in health-benefitting behavior also needs to be taken into account [7]. Besides increasing and optimizing the number of recommendations, participants need to understand these recommendations. Further, they need to be willing to take action to follow these recommendations. In the future, this could be evaluated in the biannual visits to the follow-up clinic.

Limitations: In this study, only the participants that responded to the invitation were included. During the inclusion period, more than the 204 participants reported in this study were invited for an interdisciplinary follow-up clinic. We do not know how many did not keep appointments, nor if the participants in this study might be a subgroup and therefore form some bias. Nevertheless, in this study, every person who participated in the follow-up clinic and gave consent was included in the study which makes the results well generalizable.

The current study used the classification for the recommendations as used in a previous retrospective study, to make a comparison possible. Thus, the choice of not all subjective SHCs indicated in the questionnaire could directly be linked to a specific recommendation, nor were there separate categories for the recommendation regarding the extra conducted measurements.

There might be a possibility that the questionnaire is answered differently by participants with different educational levels. We did not ask for this, so we were not able to control for that.

Lastly, during the debriefing of the follow-up clinic, the outcomes of the recommendations were not noted by the same person every time. This could have led to one person classifying a certain recommendation in a different category than someone else.

## 5. Conclusions

This study shows that adding a questionnaire prior to an interdisciplinary follow-up clinic for participants with SCI helps to increase the number of recommendations regarding SHCs. This questionnaire seems to prompt participants to think about the subjects important to discuss with the team and provides specific information to the professionals. Further, the extra physical measurements reveal, on average, high systolic blood pressure, which needs extra attention in future research. This addition seems valuable for indicating potential risks. Participants of the adapted design of the interdisciplinary follow-up clinic were very satisfied with this and about the way their questions were answered.

By continuing to evaluate and optimize this interdisciplinary follow-up clinic and giving personalized recommendations, we aim to improve daily life of participants of these clinics, to ultimately prevent or solve SHCs and to support them with aging in a healthy way. This is especially important to reverse the decrease in the number of recommendations regarding bladder problems. Future research could focus more on the preventive value of the interdisciplinary follow-up clinic by comparing the people with SCI that do not attend interdisciplinary follow-up clinics with a matched group of those who do and follow both groups over time. In addition, information about educational level could be collected. Further, we need more studies on the evolvement of blood pressure and how this can be measured best for people with SCI.

## Figures and Tables

**Table 1:** Characteristics of the participants of the interdisciplinary follow-up clinic.

Characteristics	All Participants (N = 195)	Participants in Both Designs (N = 139)
	*n* (%)/Mean (SD), Range	*n* (%)/Mean (SD), Range
Sex female/male	51 (26)/144 (74)	38 (27)/101 (73)
Age (years)	55.6 (14.0), 18–92	56.4 (13.4), 24–92
Time since injury (years)	20.9 (14.8), 2–67	23.8 (14.0), 5–67
Spinal cord injury characteristics		
Cause		
Traumatic	143 (73)	108 (78)
Vascular	12 (6)	8 (6)
Infection	16 (8)	9 (7)
Oncology	11 (6)	5 (4)
Other non-traumatic	8 (4)	6 (4)
Other/unknown	5 (3)	3 (2)
Height		
Cervical	67 (34)	50 (36)
Thoracic	113 (58)	80 (58)
Lumbar	15 (8)	9 (6)
Completeness		
AIS A	127 (65)	96 (69)
AIS B	24 (12)	13 (9)
AIS C	24 (12)	17 (12)
AIS D	18 (9)	11 (8)
Unknown/missing ^#^	2 (1)	2 (1)

^#^ Some participants acquired the SCI many years ago, before standardized measuring of level and completeness, resulting in two missing AIS scores.

**Table 2:** Total number of recommendations given in the initial (*n* = 523) and the adapted (*n* = 628) design of the follow-up clinic.

Recommendations	Preventive to Be Applied at Home		Internal Treatment		External Treatment		(Medication) Prescription		Total (%)	
	Initial	Adapted	Initial	Adapted	Initial	Adapted	Initial	Adapted	Initial	Adapted
Medical intercurrent	61	35	17	4	49	14 *	12	9	139 (27)	62 (10) *
Skin problems	14	24	3	6	2	2	3	0	22 (4)	32 (5)
Spasm	9	6	4	2	0	1	4	6	17 (3)	15 (2)
Pain	11	12	12	3	6	6	6	3	35 (7)	24 (4)
Bladder problems	28	19	2	0	25	6 *	5	1	60 (12)	27 (4) *
Bowel problems	24	16	3	4	1	1	8	4	36 (7)	25 (4)
Lung problems	10	42 *	2	4	1	5	0	2	13 (3)	53 (8) *
Functioning	35	73 *	18	22	10	14	1	1	64 (12)	110 (18)
Social	6	27 *	3	10	2	0	0	0	11 (2)	37 (6) *
Psychological	5	23 *	2	5	6	3	0	0	13 (3)	31 (5)
Devices	18	79 *	23	32	19	5	9	3	69 (13)	119 (19) *
Splints	1	12	6	7	3	1	4	0	14 (3)	15 (2)
Seating advice	12	49 *	18	25	0	4	0	0	30 (6)	78 (124) *

(N = 139, * *p* < 0.001 according to a paired samples *t*-test).

**Table 3:** Conducted measurements of the participants who visited both designs.

Measurements	N	Initial Design	N	Adapted Design
		*n* (%)/Mean (SD), Range		*n* (%)/Mean (SD), Range
Pressure injuries or wounds present	136	30 (22)	139	42 (30)
Weight in kg	105	76.9 (8.9), 32.6–116.0	128	81.2 (16.4), 33.8–132.6
FVC, % of predicted value	101	72.9 (23.7)	86	67.4 (21)
PEF, % of predicted value	102	66.5 (22.7)	85	65.7 (30)
FEV_1_, % of predicted value	101	73.0 (23.1)	85	67.8 (23.1)
Oxygen saturation in SpO2%			133	97.5 (1.9)
Systolic/diastolic blood pressure in mm Hg			137	136/80 (26/13)
Pulse rate in beats per minute			136	74 (14.9)

**Table 4:** Mean scores on the validated scales used in the assessment and their correlation with the total number of recommendations.

Measurements	N	Mean (SD), Range	Correlation with Total Recommendations
SCI-SCS (16 items, range 0–48)	176	13.2 (7.3), 0–41	0.37 **
QoL-BDS total (4 items, range 0–10)	136	6.9 (1.5), 0–10	−0.12
QoL-BDS life as whole (range 0–10)	137	7.0 (1.7), 0–10	−0.13
QoL-BDS physical health (range 0–10)	136	6.4 (1.9), 0–10	−0.19 *
QoL-BDS psychological health (range 0–10)	136	7.1 (1.8), 0–10	−0.07
QoL-BDS social life (range 0–10)	136	7.1 (1.8), 0–10	−0.02
PHQ-2 (2 items, range 0–6)	175	0.91 (1.2), 0–6	0.08
GAD-2 (2 items, range 0–6)	103	0.91 (1.3)/0–5	0.21 *

Abbreviations: SCI-SCS, spinal cord injury secondary condition scale; QoL-BDS, international quality of life basic data set; PHQ-2, Patient Health Questionnaire-2; GAD-2, Generalized Anxiety Disorder-2. * *p* < 0.01, ** *p* < 0.05.

## Data Availability

The data presented in this study are available on request from the corresponding author.
